# Dysbiosis of the Oral Ecosystem in Severe Congenital Neutropenia Patients

**DOI:** 10.1002/prca.201900058

**Published:** 2020-03-03

**Authors:** Egija Zaura, Bernd W. Brandt, Mark J. Buijs, Gülnur Emingil, Merve Ergüz, Deniz Yilmaz Karapinar, Burç Pekpinarli, Kai Bao, Georgios N. Belibasakis, Nagihan Bostanci

**Affiliations:** ^1^ Department of Preventive Dentistry Academic Centre for Dentistry Amsterdam (ACTA) Vrije Universiteit Amsterdam and University of Amsterdam 1600NP Amsterdam The Netherlands; ^2^ Department of Periodontology School of Dentistry Ege University İzmir 35100 Turkey; ^3^ Department of Pediatric Hematology School of Medicine Ege University İzmir 35100 Turkey; ^4^ Department of Pediatrics School of Dentistry Ege University İzmir 35100 Turkey; ^5^ Division of Oral Diseases Department of Dental Medicine Karolinska Institutet Huddinge 14104 Sweden

**Keywords:** chemokines, congenital neutropenia, cytokines, gingival crevicular fluid, inflammation, microbiota, saliva

## Abstract

**Purpose:**

To decipher the underlying immunological mechanisms in predisposition to oral microbial dysbiosis in severe congenital neutropenia (SCN) patients.

**Experimental Design:**

Ten SCN patients (5–23 years old) and 12 healthy controls (5–22 years old) are periodontally examined and provided saliva, subgingival plaque, and gingival crevicular fluid (GCF) samples. The SCN patients received oral hygiene therapy and are re‐evaluated after 6 months. Antimicrobial peptides HPN1‐3 and LL‐37 are assessed in saliva by ELISA. Concentration of 30 cytokines is measured in saliva and GCF by human 30‐plex panel, while bacterial profiles of saliva and subgingival plaque are assessed using 16S rDNA amplicon sequencing.

**Results:**

There is no significant difference in salivary HPN1‐3 and LL‐37 concentration between the SCN patients and controls. At baseline, clinical, immunological, and microbiological parameters of the patients are indicative of oral ecological dysbiosis. The SCN patients have significantly higher bleeding on probing (BOP)%, GCF volume, and cytokine levels, high bacterial load with low bacterial diversity in saliva. The associations between the microbiome and immunological parameters in the SCN patients differ from those in the healthy individuals.

**Conclusions and Clinical Relevance:**

SCN patients have a dysregulated immune response toward commensal oral microbiota, which could be responsible for the observed clinical and microbiological signs of dysbiosis.

## 1 Introduction

Severe congenital neutropenia (SCN) is a rare genetic disorder, usually diagnosed during the first months of life and associated with severe recurrent infections and persistently low neutrophil counts.^[^ ^1^ ^]^ Its prevalence has been estimated around 3–8.5 cases per million individuals.^[^ ^2^ ^]^ The condition was first described by Swedish pediatrician Rolf Kostmann in 1950s as a hereditary infantile agranulocytosis and is therefore also recognized as Kostmann syndrome.^[^ ^3^ ^]^

In 1990, a therapy with granulocyte colony‐stimulating factor (G‐CSF), found to restore blood neutrophil counts in most of the SCN patients to physiological levels, was introduced.^[^ ^4^ ^]^ Although the G‐CSF therapy has led to increased life expectancy and quality of life in the affected individuals, they still remain prone to infections, and the SCN is associated with poor periodontal health.^[^ ^5^, ^6^, ^7^ ^]^

Dysregulation of the homeostasis of neutrophils is proposed to influence periodontal health,^[^ ^8^ ^]^ making the SCN patients an interesting group for addressing the etiology of oral dysbiosis in relation to periodontal diseases. To date, only limited, cross‐sectional information on oral microbiota of the SCN patients is available, for instance on salivary^[^ ^9^ ^]^ or subgingival^[^ ^10^ ^]^ microbiome, while a broad evaluation at the level of the ecosystem is lacking.

In this study, we aimed at exploring the oral ecosystem of young SCN patients receiving G‐CSF therapy. For this, we compared oral health status, oral microbiome, and immunological profiles of these individuals before and after oral hygiene therapy, with those of the healthy controls.

## 2 Results

### 2.1 Study Population Characteristics

Ten SCN patients (9 females, 1 male), aged 5–23 years from different parts of Turkey were enrolled (**Table** **1**) and compared with 12 healthy gender‐matched controls (aged 5–22 years, 11 females, 1 male). There was no significant difference in age between the groups. None of the study subjects were either current or former smoker. Detailed description of the diagnosis, disease severity, mutations, and medical analyses of the patients is provided in the Supplementary file. All patients had a history of recurrent infections, the frequency of infections varied among patients with a median of 6 times a year (range 4–8 times a year) before the G‐CSF therapy and twice a year (0–4) after the G‐CSF treatment was started. Before the G‐CSF therapy, median absolute neutrophil counts (ANC) were 320 (112–440) x10^6^/l (Table S1, Supporting Information). The G‐CSF therapy was adjusted to raise neutrophils to approximately 1000 × 10^6^/l.

**Table 1:** Age, diagnosis, medication, and genetic findings of the congenital neutropenia patients of the study

Patient	Gender/age	Weight [kg]	Age first symptoms/neutropenia diagnosis	Follow‐up duration	ANC/severity of neutropenia	Family history	G‐CSF duration/dose	Gene mutation	Consanguinity between parents
101	F/8 y 8 mo	28	7/15 mo	7 y 5 mo	Very severe	No	For 2 y	Negative^b^	No
102	F/7 y 7 mo	21	4/19 mo	3 y 7 mo	Very severe	No	For 2 y/5 μg kg^−1^/3 d per week	bi‐allelic CSF3R mut(+)	Yes, 1st degree cousin marriage
103	F/7 y 3 mo	20	11/29 mo	3 y 8 mo	Severe	No	For 2 y	Negative^b^	No
104	M/22 y 8 mo	62	5/41 mo	13 y 8 mo	Severe	No	For 12 y 5 μg kg^−1^/3 d per week	Homozygous HAX1(+)	Yes, 1st degree cousin marriage
105	F/23 y 4 mo	55	4/11 mo	9 y 4 mo	Very severe	No	For 12 y 9 mo 5 μg kg^−1^/2 d per week	Homozygous HAX1(+)	Yes, 1st degree cousin marriage
106	F/7 y	32	4/19 mo	3 y 6 mo	Very severe	No	For 2 y 3 mo 3–5 μg kg^−1^/3–5 d per week	bi‐allelic CSF3R mut(+)	No
107	F/10 y	33	3/17 mo	8 y 7 mo	Severe	No	For 8 y 8 mo 5 μg kg^−1^/3–4 d per week	GSDtype1b	No
108	F/5 y 9 mo	24	1/27 mo	3 y 6 mo	Very severe	No	For 4 y 3 mo 5 μg kg^−1^/5 d per week	Heterozygous ELANE mut(+)	No
109	F/15 y 11 mo	52	2/36 mo	12 y 6 mo	Very severe	Yes^a^	For 11 y 8 mo 5 μg kg^−1^/3 d per week	Homozygous HAX1(+)	Yes, 1st degree cousin marriage
110	F/7 y	26	2/2 mo	7 y	Very severe	Yes^a^	For 6 y 11 mo 5 μg kg^−1^/3 d per week	Homozygous HAX1(+)	Yes, 1st degree cousin marriage

Year, y; month, mo;

a patients P9 and P10 were sisters; their cousin had the same mutation;

b ELANE, HAX1, G6PC3, JAGN1, SBDS, and CSF3R mutations were all screened and found to be negative.

### 2.2 Oral Clinical Findings of the Study Population

**Figure 1:**
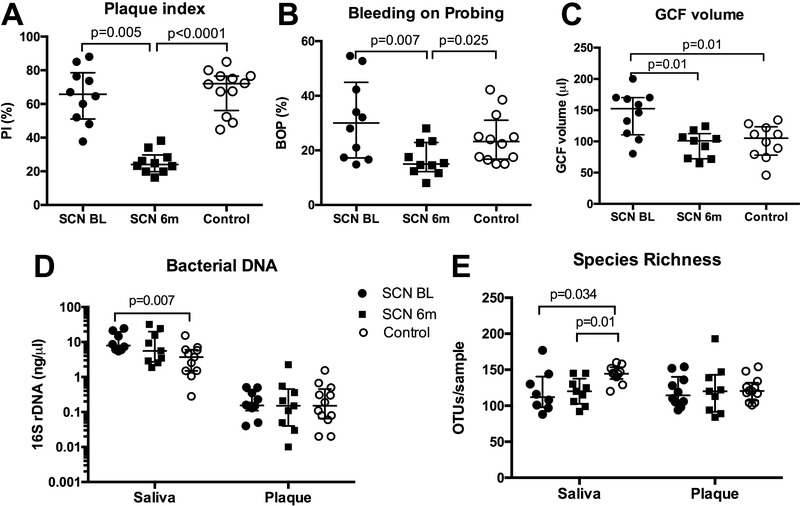
A) Plaque index, B) bleeding on probing (BOP%), C) volume of gingival crevicular fluid (GCF), D) concentration of bacterial DNA, and E) bacterial species richness in unstimulated saliva and subgingival plaque samples collected in SCN patients at baseline (SCN BL), after a 6‐month follow‐up (SCN 6m) and in healthy controls (Control). Connectors indicate statistically significant differences (*p* < 0.05, Mann–Whitney test for independent samples and Wilcoxon signed ranks test for paired samples).

The number of decayed missing filled teeth (DMFS) or dmft in patients did not differ from the controls (*p* > 0.05) (Table S2, Supporting Information). None of the patients had oral lesions. Both groups had similar periodontal probing depth (PPD), clinical attachment level (CAL), bleeding on probing (BOP) (%), plaque index (PI, %) at baseline (*p* > 0.05). At the 6 month‐recall of the SCN patients, there was a statistically significant improvement in BOP (%) and in PI (%) compared to the baseline and compared to the control group (**Figure** **1**A,B).

**Clinical Relevance**

Although individuals with severe congenital neutropenia (SCN) receiving granulocyte colony‐stimulating factor (G‐CSF) therapy gain (nearly) physiological levels of neutrophils, they are still prone to infections and periodontal diseases. Due to the involvement of neutrophils in periodontal health, the SCN patients are an interesting group for addressing the etiology of oral dysbiosis in relation to periodontal diseases. Here we investigated the oral microbiome in relation to oral health status and immunological profiles of SCN patients before and after oral hygiene therapy, in comparison with healthy controls. At baseline, clinical, immunological, and microbiological parameters of the patients were all indicative of oral ecological dysbiosis. After the oral hygiene intervention, both clinical and immunological parameters showed partial recovery toward a healthy state, while salivary microbial profiles remained distinct from the controls. Moreover, the associations between the microbiome and immunological parameters in the SCN patients were distinct from those of controls and remained such after the improvement of their clinical oral health status. We conclude that SCN patients with normalized neutrophil counts due to G‐CSF therapy, have a dysregulated immune response toward commensal oral microbiota, which could be responsible for the observed clinical and microbiological signs of dysbiosis in these individuals.

### 2.3 Salivary Antimicrobial Peptides and Cytokine Profiles

There were no statistically significant differences between the test and the control group or between the baseline and a 6‐month recall in the concentration of salivary peptides HPN1‐3 and LL‐37 (Table S3, Supporting Information).

Among the cytokines measured in saliva (Table S3, Supporting Information), four cytokines were significantly higher in the SCN patients at baseline and at the 6‐month recall compared to the control group, and another four cytokines decreased significantly between the baseline and the 6‐month recall visit in the patients.

**Figure 2:**
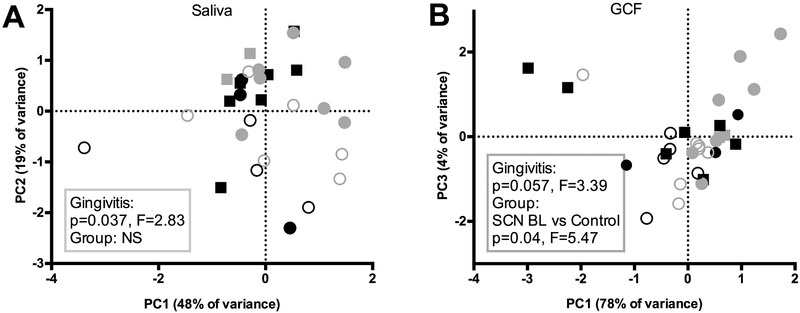
Principal component analysis (PCA) plots of immunological parameters measured in A) saliva and in B) GCF of SCN patients (filled symbols) and controls (open circles) by presence of gingivitis. Samples collected from subjects with gingivitis (>20% of gingival bleeding) are depicted in gray; without gingivitis, in black; SCN baseline samples, dots; SCN 6‐month follow‐up samples, filled squares. Boxes indicate PERMANOVA results by gingivitis and by group. NS, not significant (*p* > 0.05).

Next, we assessed the differences in the immunological profiles using principal component analysis (PCA) on all 30 cytokines in saliva (**Figure** **2**A). There was no statistically significant pattern by group, but by the presence of gingivitis (defined as gingival bleeding above 20%).

### 2.4 Gingival Crevicular Fluid Volume and Cytokine Profiles

At baseline, the average volume of gingival crevicular fluid (GCF) per sampled site in the SCN patients was significantly higher than in the control group or at the 6‐month follow‐up (Figure 1C).

At baseline, 25 of the 30 cytokines were at a significantly higher concentration in the GCF samples of the SCN‐patients compared to the controls (Table S4, Supporting Information). Among these 25, the concentration of four cytokines (IL‐1 β, MCP‐1, MIG, IP‐10) decreased significantly between the baseline and the 6‐month follow‐up, while a single cytokine (IL‐4) that did not differ from the controls, decreased significantly between the baseline and the 6‐month follow‐up in the SCN patients.

Immunological profile analysis by PCA and PERMANOVA depicted a significant difference between the GCF samples collected at the baseline in the SCN patients versus controls (Figure 2B).

### 2.5 Oral Microbiome Composition

Microbial DNA concentration was significantly higher in the saliva samples collected from the SCN patients at baseline compared with the control subjects, while no difference was found in the plaque samples (Figure 1D).

After clustering, on average, 20 484 reads (SD 4230, min 11 870, max 29 284) were obtained per sample. To normalize for unequal sequencing depth, the dataset was randomly subsampled at 11 800 reads per sample. The normalized dataset contained 346 operational taxonomic units (OTUs) that were classified into 16 phyla and 150 genera or higher taxa, with phylum Firmicutes dominating the dataset (38% of all reads), followed by Bacteroidetes (20%), Proteobacteria (18%), Fusobacteria (14%), Actinobacteria (9%), TM7 (0.6%), and Spirochaetae (0.1%), together accounting for 99.9% of the reads. The genus *Streptococcus* with 21% of the reads was the most predominant genus, followed by *Prevotella* (13%), *Veillonella* (10%), *Leptotrichia* (9.7%), *Neisseria* (7%), *Fusobacterium* (4%), *Actinomyces* (4%), *Haemophilus* (4%), and *Rothia* (3.4%) (Figure S1, Supporting Information).

Saliva of the control subjects had significantly higher species richness compared to saliva of the SCN patients both at baseline and after the 6‐month follow‐up (Figure 1E) and a significantly higher Shannon diversity index compared to saliva of the SCN patients after the 6‐month follow‐up (*p* = 0.001). However, no differences in microbial diversity were observed in subgingival plaque.

**Figure 3:**
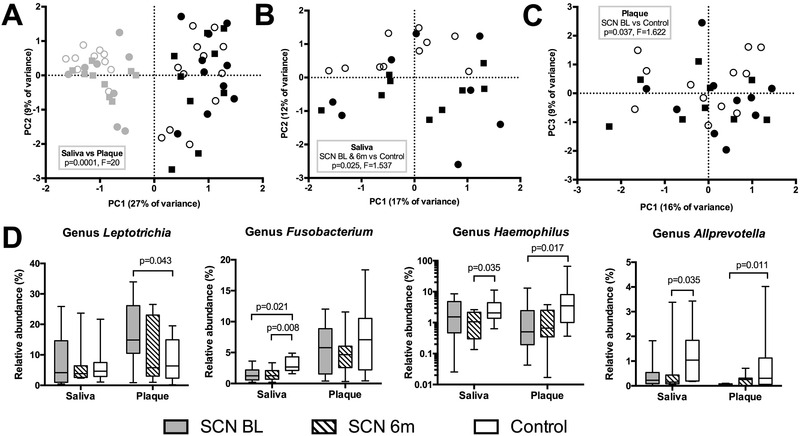
A) PCA plots of saliva (gray) and subgingival plaque (black) samples; b) saliva samples alone and C) plaque samples alone (C) by group: dots, SCN baseline; squares, SCN 6‐month follow‐up; circles, Control group. D) Major bacterial genera that showed significant differences among the sample groups (*p* < 0.05, Mann–Whitney test) in saliva and plaque.

Microbial profiles showed strong clustering by sample type—saliva or subgingival plaque (**Figure** **3**A). Salivary microbial profiles (Figure 3B) of the SCN patients, both from baseline and the 6‐month follow‐up, differed significantly from the control samples, while subgingival plaque (Figure 3C) collected at the baseline from the SCN patients differed significantly from the control group.

The contribution of individual microbial taxa to the observed differences in microbial profiles was further investigated using linear discriminant analysis (LDA) Effect Size (LEfSe) tool, both at the OTU and at the genus or higher taxon level. At the OTU‐level, 19 OTUs discriminated between the SCN baseline and the controls, all significantly higher in the saliva samples of the controls (Figure S2A, Supporting Information). At the genus or higher taxon level, eight taxa, among which *Fusobacterium*, candidate division TM7 and Clostridiales Family XIII *Incertae Sedis*, all higher in the control samples, discriminated between the SCN baseline and the controls. After the 6‐month follow‐up, saliva samples of the SCN patients had significantly lower relative abundance of reads classified as genus *Alloprevotella*, *Fusobacterium*, and *Haemophilus* compared to the controls, while none of the genera discriminated between the baseline and the follow‐up samples (Figure 3D).

In subgingival plaque, 15 OTUs discriminated between the SCN baseline samples and the controls, with eight OTUs being significantly higher in the SCN samples (Figure S2B, Supporting Information). At the genus level, seven taxa discriminated between the SCN‐baseline samples and the controls, of which only genus *Leptotrichia* was at a significantly higher relative abundance in the plaque of the SCN patients compared to the controls (Figure 3D). After the 6‐month follow‐up, plaque of the SCN‐patients had significantly lower relative abundance of the reads classified as genus *Haemophilus* compared to the controls, and showed an increased proportion of genus *Corynebacterium* and *Acinetobacter* in comparison to the baseline samples.

### 2.6 Relation between the Immunological Parameters and Microbiome

**Figure 4:**
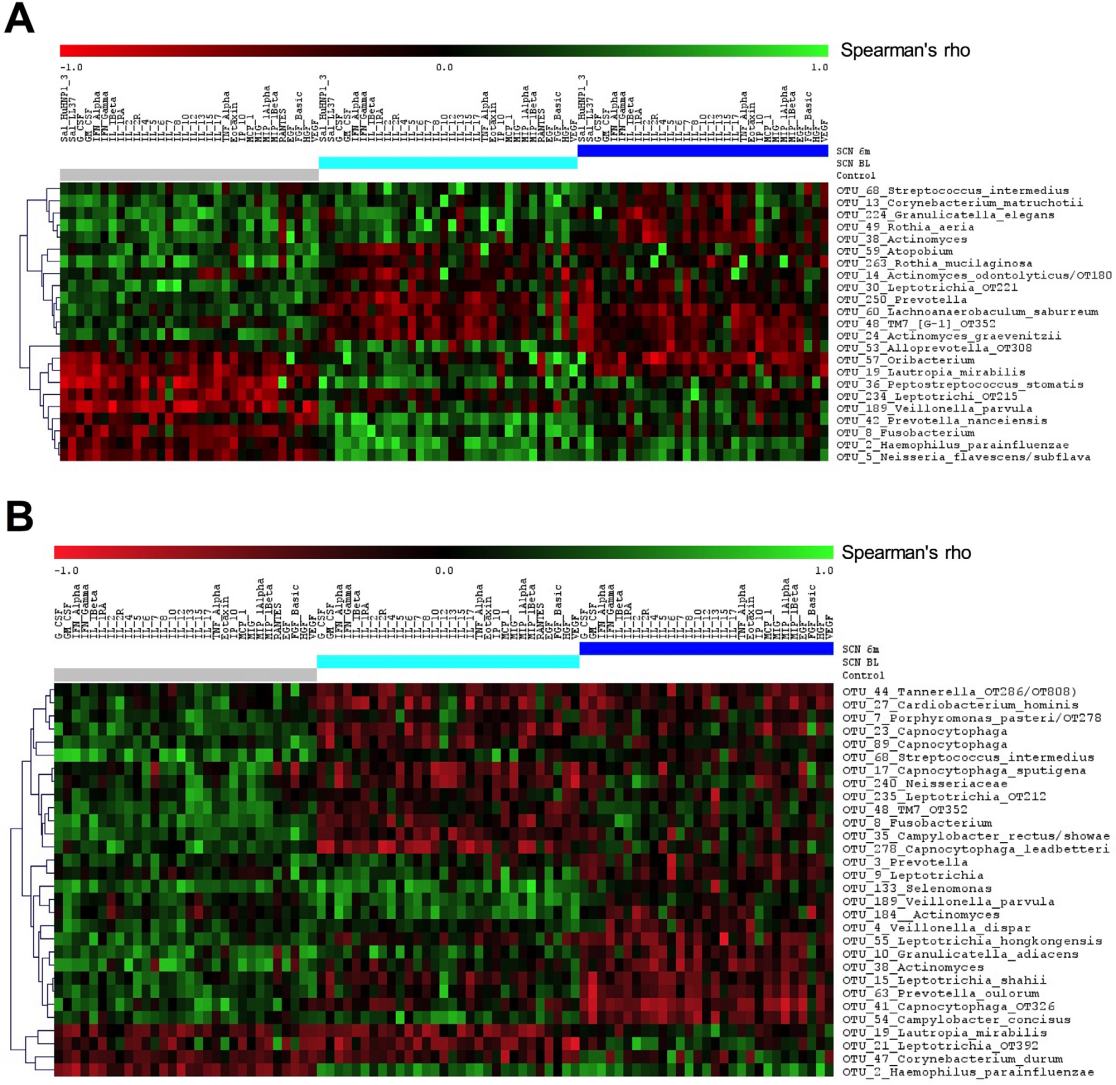
Correlation between significantly discriminatory OTUs and immunological parameters among the sample groups in A) saliva and B) subgingivally. The sample groups: Control subjects (gray), SCN patients at baseline (aqua) and SCN patients at 6‐month follow‐up (blue). In saliva, of the 42 OTUs that were present in all three groups at a min 0.1% abundance and significantly correlated with at least one immunological parameter in at least one of the groups (*p* < 0.05, Spearman's correlation), 23 OTUs significantly discriminated the groups (SAM, false discriminatory rate 0%, delta value 0.3), while in plaque these were 30 out of 53 OTUs. Green, positive; red, negative values of Spearman's correlation.

Next, we assessed if there were any associations between the immunological parameters and the microbiome. For this, each individual immunological parameter in either saliva or GCF sample of each subject was correlated with each individual OTU that belonged to the top 0.1% OTUs in the respective saliva or subgingival plaque sample. The highest number of significant correlations (134 in subgingival and 127 in salivary samples) were found in the SCN patients at baseline, followed by the control samples (118: subgingival and 95: salivary) and SCN 6‐month follow‐up samples (110: subgingival and 75: salivary) (Table S5, Supporting Information). Majority (85%) of the correlations between the OTUs and immunological parameters in the baseline saliva of the SCN patients were positive, while in subgingival plaque the most correlations (60%) were negative. In the control subjects, the opposite was observed: 60% of the correlations in saliva were negative and 87% of the subgingival correlations were positive. In both saliva and subgingival plaque, there were several OTUs that discriminated significantly among the three groups of samples in their correlation coefficient values with the immunological parameters (Figure **4**A,B). In other words, the associations between the microbiome and immunome in the SCN patients differed from those in the healthy individuals.

## 3 Discussion

To our knowledge, this is the first investigation of the oral microbiome in relation to oral health status and immunological profiles of SCN patients before and after oral hygiene therapy, in comparison with healthy controls. At baseline, clinical, immunological, and microbiological parameters of the patients were all indicative of oral ecological dysbiosis. After the oral hygiene intervention, both clinical and immunological parameters showed partial recovery toward a healthy state, while salivary microbial profiles remained distinct from the controls. Moreover, the associations between the microbiome and immunological parameters in the SCN patients were distinct from those of controls and remained such after the improvement of their clinical oral health status.

All SCN patients participating in our study were exposed to G‐CSF therapy to raise the neutrophil counts to physiological levels. Previous pilot‐scale studies (4–6 patients) reported deficiency in innate immune components released by neutrophils—antimicrobial peptides defensin HNP1‐3 and cathelin LL‐37—in saliva and plasma of the SCN patients, concluding that G‐CSF therapy reversed neutropenia but did not correct the functional deficiency of neutrophils.^[^ ^5^, ^6^, ^11^ ^]^ This finding however was not confirmed in our study: saliva of the SCN patients and controls had comparable concentrations of the two antimicrobial peptides, indicating that G‐CSF therapy in our cohort had led to a rise in functional neutrophils, at least regarding these molecules. This could be due to the fact that only a single patient in our cohort was identified with heterozygous ELANE mutation of the gene associated with expression of neutrophil elastase—ELA2, linked with the deficiency in neutrophil functioning in the SCN patients.^[^ ^6^ ^]^ The most common mutation in our cohort was homozygous HAX1 mutation, which corresponds to the most prevalent SCN‐associated mutation in Turkey.^[^ ^11^ ^]^ It is possible that in the absence of ELANE mutations, G‐CSF treatment recovers several neutrophil‐derived antibacterial proteins via increased metabolic burst associated with phagocytosis.^[^ ^13^ ^]^

At baseline, the oral health status of the SCN patients was comparable to that of the controls, yet the former group responded to the same amount of dental plaque with a higher pronounced production of GCF. Reversely, at the 6‐month follow‐up the amount of plaque was significantly lower in the SCN patients than in the controls, yet there were no differences in GCF volume levels. It was therefore not surprising to detect higher concentrations of selective cytokines (e.g., IL‐1β, IL‐2, IL‐4, EGF, and HGF) in the GCF of the SCN patients, compared to the controls, indicating an inherent perturbation of inflammatory networks within the oral milieu of these patients.

Based on the findings above, we anticipated that the SCN patients would interact differently with their oral microbiome than healthy subjects. In agreement with the previously published work on a different cohort of young Turkish SCN patients,^[^ ^9^ ^]^ we found lower bacterial diversity in saliva of the patients in comparison to the controls. Surprisingly though, these samples had higher bacterial DNA concentration (equivalent to bacterial cell counts) than the controls—a finding which had not been assessed in the study above. Most likely explanation for high bacterial counts in saliva is due to deficient innate immune system, characteristic for SCN patients.^[^ ^4^, ^6^ ^]^

The saliva samples from the SCN patients in the 2019 study by Topcuoglu et al. had higher proportion of the genera *Streptococcus* and *Granulicatella* than the controls—both being saccharolytic taxa, most likely reflecting a higher caries prevalence in the SCN patients compared to their controls.^[^ ^9^ ^]^ No difference in caries nor taxa potentially associated with caries were detected in our study population.

Regarding subgingival plaque, the only taxon that was found at a higher proportion in the SCN patients belonged to genus *Leptotrichia*. These are slow‐growing, non‐motile Gram‐negative, facultative or strictly anaerobe commensal bacteria that recently have been considered as opportunistic causes of human infections (e.g., pneumonia, mucositis, sepsis) especially in immunocompromised hosts such as neutropenia patients.^[^ ^14^ ^]^

Simultaneously acquired samples for microbial and immunological data provided us with unique opportunity to relate these two multivariate datasets with each other. This way we observed that the SCN patients had a fingerprint of the associations between the immunological and microbiological parameters that was distinct from the controls, and that salivary and subgingival associations were distinct from each other. For instance, in healthy subjects, both salivary and subgingival immunological parameters correlated negatively with oral commensal species—*Haemophilus parainfluenzae*, while in the SCN patients a strong positive correlation was observed, indicating dysregulated pro‐inflammatory immune response of SCN patients to commensal microbiota. On the other hand, subgingival plaque microbiota of the SCN patients related negatively with the majority of the immunological parameters, indicative of subversion of the immune response, while in healthy subjects nearly all associations were positive and thus indicative of an activated immune response. Mechanistic studies on commensal taxa and immune response from hosts with different phenotypes should be performed to decipher the current observations their clinical relevance.

One of the limitations of this study is its small sample size. Low prevalence of SCN precluded us from obtaining a larger group of cases. This is a common issue in studies on genetically rare conditions. Additionally, all but one of the subjects were females, biasing the results toward one gender and precluding generalization of the current findings. For that, a larger group with higher proportion of males with SCN should be studied. Finally, the prophylactic exposure to a 5‐day course of antibiotics, starting a day before the clinical examination and the collection of the subgingival plaque and GCF samples was performed, might have influenced the study outcomes. Since collection of saliva is non‐invasive, these samples, used for antimicrobial peptide and cytokine assessment and microbiome analyses, were collected before the start of the antibiotic administration, thereby avoiding potential bias by the prophylaxis. It should also be noted that all SCN patients have experienced recurrent infections and have been exposed to antibiotics since their early childhood. This might have contributed to the observed differences in salivary microbiome composition and bacterial diversity between the cases and the controls.

In conclusion, SCN patients with normalized neutrophil counts due to G‐CSF therapy, have a dysregulated immune response toward commensal oral microbiota, which could be responsible for the observed clinical and microbiological signs of dysbiosis in these individuals.

## 4 Experimental Section

Full materials and methods are described in the Supplementary file. The study protocol was approved by the Ethics Committee of Ege University, Izmir, Turkey (B.30.2.EGE.0.20.05.00/EY/15‐9/1). A written informed consent was obtained from all participants. Ten SCN patients and 12 systemically healthy controls were included in the study. The medical assessment and SCN diagnosis of the patients was performed as described previously.^[^ ^12^ ^]^ The control group consisted of systemically healthy individuals attending the clinic of the Department of Paediatric Dentistry.

##### Clinical Dental and Periodontal Examinations

The SCN patients were referred to the dental clinics at the Department of Periodontology for oral health screening. During this visit, dental X‐rays were taken, saliva samples were collected, and patients filled a questionnaire regarding oral lesions, the frequency of dental visits, use of antibiotics, bleeding on brushing.

Clinical oral examination and sample collection from the SCN patients was performed at the second visit, 1‐day after the start of antibiotic prophylaxis (amoxicillin and clavulanic acid, 30–50 mg kg^−1^ d^−1^ for 5 days). Both SCN patients and controls underwent assessment of probing pocket depth (PPD), clinical attachment level (CAL), presence of plaque and presence of bleeding on probing (BOP). All subjects received scaling and oral hygiene instructions. In the SCN group, the clinical examination and sample collection was repeated after 6 months (6‐month follow‐up visit), again 1‐day after the start of the antibiotic prophylaxis.

##### Sample Collection

Whole unstimulated saliva sample was collected by passive drooling. GCF samples were obtained from mesiobuccal aspects of first molars as described previously.^[^ ^15^ ^]^ Subgingival plaque samples were collected from the same sites as GCF, as described earlier.^[^ ^16^ ^]^ All samples were stored at −80 °C.

##### 16S rDNA Amplicon Sequencing of Subgingival Plaque and Saliva Samples

Full description of sample processing and amplicon sequencing is described in the Supplementary file. In brief, DNA was extracted from saliva and subgingival plaque using bead‐beating procedure and the Mag MiniKit (LGC Genomics, Berlin, Germany, Mag mini kit). Bacterial DNA concentration was determined by16S rRNA gene specific qPCR.^[^ ^17^ ^]^ V4 hypervariable region of the 16S rRNA gene was amplified,^[^ ^18^ ^]^ the amplicons were pooled equimolarly, purified and paired‐end reads were generated using the Illumina MiSeq platform (Illumina, Inc., San Diego, CA). The reads were processed into OTUs as described previously.^[^ ^19^ ^]^ The most abundant sequence of each OTU was classified using the RDP classifier^[^ ^20^ ^]^ and HOMD version 14.51.^[^ ^21^ ^]^

##### Assessment of Salivary Antimicrobial Peptides

Saliva samples were centrifuged for 30 min at 1000 rpm at 4 °C (Eppendorf Thermomixer). ELISA Hu‐HNP1‐3 and ELISA Hu‐LL‐37 (Hycult Biotech) were performed according to the instructions of the manufacturer.

##### Cytokine Profiling by Multiplex Assay

The cytokines in GCF and saliva were quantified using the cytokine 30‐Plex panel (Novex, ThermoFisher Scientific, USA) as described previously.^[^ ^22^ ^]^ The panel consisted of nineteen cytokines: G‐CSF, GM‐CSF, IFN‐α, IFN‐γ, IL‐1β, IL‐1RA, IL‐2, IL‐2R, IL‐4, IL‐5, IL‐6, IL‐7, IL‐8, IL‐10, IL‐12 (p40/p70), IL‐13, IL‐15, IL‐17, TNF‐α; seven chemokines: Eotaxin, CXCL10 (IP 10), MCP‐1, MIG, MIP‐1α, MIP‐1β, RANTES, and four growth factors: EGF, FGF‐basic, HGF, VEGF.

##### Statistical Analyses

Detailed statistical analyses are described in the Supplementary file. In brief, differences in univariate variables were assessed using appropriate tests in SPSS version 25. Multivariate data was analyzed using principal coordinate analysis (PCA) and permutational analysis of variance (PERMANOVA) with Bray–Curtis similarity, using PAST software.^[^ ^23^ ^]^ Discriminatory OTUs or genera were identified using LDA effect size (LEfSe) biomarker discovery tool.^[^ ^24^ ^]^

The associations between the immunological parameters and microbiome were tested using Spearman correlation in R (version 3.6.0). Significant differences among the groups were assessed using multiclass significance analysis of microarrays (SAM), TM4, MEV version 4.9.0.^[^ ^25^ ^]^

##### Associated Data

The raw 16S rDNA sequence data is available at NCBI short read archive (SRA) under bioproject ID PRJNA564282.

## Conflict of Interest

The authors declare no conflict of interest.

## Author Contributions

E.Z. contributed to data analyses, drafted, and finalized the manuscript; B.W.B. contributed to data analyses and critically revised the manuscript; M.J.B. contributed to data acquisition and critically revised the manuscript; G.E. contributed to the design of the study, drafted, and critically revised the manuscript; M.E. contributed to patient care, patient selection, and clinical data collection; D.Y.K. contributed to patient care, patient selection, and clinical data collection; B.P. contributed to patient care, patient selection, and clinical data collection; K.B. contributed to sample preparation, data acquisition, and critically revised the manuscript; G.N.B. contributed to study design, data analysis, and critically revised the manuscript; N.B. conceived and designed the study, contributed to data analyses, drafted and finalized the manuscript; all authors gave their final approval and agree to be accountable for all aspects of the work.

## Supporting information

Supporting Information
