# Supplementary material for: Dysbiosis of the Oral Ecosystem in Severe Congenital Neutropenia Patients
Source: Proteomics Clin Appl. 2020 Mar 3;14(3):1900058. doi: 10.1002/prca.201900058 (PMC7317524; doi:10.1002/prca.201900058)
Supplement: Supplementary file 1 — Supporting Information [file PRCA-14-1900058-s001.docx]

**Supplementary file:**

**Dysbiosis of the oral ecosystem in severe congenital neutropenia patients**

Egija Zaura, Bernd W. Brandt, Mark J. Buijs, Gülnur Emingil, Merve Ergüz, Deniz Yilmaz Karapinar, Burç Pekpinarli, Kai Bao, Georgios N. Belibasakis, Nagihan Bostanci

**Full materials and methods**

Ten patients diagnosed with severe congenital neutropenia (SCN) and 12 systemically healthy controls were recruited for the study. The study protocol was approved by the Ethics Committee of Ege University, Izmir, Turkey (B.30.2.EGE.0.20.05.00/EY/15-9/1). The design and the aim of this study were explained in detail prior to recruitment and a written informed consent was obtained from all participants.

*Inclusion and exclusion criteria for patients with congenital neutropenia*

The medical assessments were performed at the Pediatric Hematology Department, Ege University as described previously (Yılmaz Karapınar et al. 2019). Briefly, routine laboratory tests included complete blood counts (CBC), absolute neutrophil count (ANC), immunoglobulin levels (G, A, M and E), bone marrow aspirate or biopsy, as well as abdominal ultrasonography/cardiac echocardiography to exclude renal and cardiac abnormalities. Additionally, plasma and urine amino acids, and vitamin B12 and folate assays were carried out to eliminate organic acidemia and megaloblastic neutropenia, respectively. To exclude myelodysplastic changes in bone marrow, chromosomal abnormalities were evaluated using cytogenetic and fluorescent in situ hybridization methods. Exclusion criteria included drug, infection related or chemotherapy-induced neutropenia, thrombocytopenia, myelodysplastic syndrome, aplastic anemia, known HIV infection, or other hematological disease and known immune diseases such as rheumatoid arthritis, systemic lupus, and autoimmune neutropenia. Patients who had active infection and life-threatening low neutropenia levels, transient neutropenia, autoimmune neutropenia, spontaneous remission of neutropenia were also excluded.

*Inclusion and exclusion criteria for the* *control group*

Twelve systemically healthy age and sex matched individuals who were attending the Department of Paediatric Dentistry clinics for regular dental check-ups were recruited as a control group. The exclusion criteria included a history of periodontal treatment, or use of antibiotics or any medications or anti-inflammatory drugs within the past six months.

*Clinical dental and periodontal examinations*

Diagnosis of SCN in patients was made by an expert (DYK) on the basis of clinical and hematologic findings. These ten patients were referred to the dental clinics at the Department of Periodontology, School of Dentistry, Ege University for oral health screening (visit 1). During this visit, the participants were asked to fill a questionnaire including questions about presence of mouth sores/ulcerations, the frequency of regular dental visits, use of antibiotics, presence/absence of bleeding on brushing. The dental X-rays were also taken and whole saliva samples were collected.

Both SCN patients and control subjects underwent a full-mouth periodontal examination as well as clinical sample collection performed by the same calibrated examiner (ME). For the SCN patients, this was done at the second visit, one-day after the start of antibiotic prophylaxis (amoxicillin and clavulanic acid, 30-50 mg/kg body weight/day, for five days).

Clinical periodontal parameters included recording of probing pocket depth (PPD), clinical attachment level (CAL), presence of plaque and presence of bleeding on probing (BOP). All measurements were recorded at six sites on each fully erupted tooth present using a Williams periodontal probe. All subjects received scaling and oral hygiene instructions (ME). In the SCN group, the clinical examination and sample collection was repeated after 6 months (6-month follow-up visit), one-day after the start of the antibiotic prophylaxis as above.

*Collection and processing of saliva samples*

The participants were requested not to drink or eat and omit any oral hygiene procedures including brushing, floosing or mouth rinsing up to 2 h prior to sampling. Whole unstimulated saliva sample was collected by passive drolling into sterile 50-ml polypropylene tube for ten minutes. Obtained saliva samples were immediately frozen and stored at -80°C until further analysis.

*Collection and processing of gingival crevicular fluid samples*

GCF samples were taken from mesiobuccal aspects of first molars in each participant as described earlier (Bostanci et al. 2013). Prior to GCF sampling, the supragingival plaque was removed from the interproximal surfaces with a sterile curette; these surfaces were dried gently by an air syringe and were isolated by cotton rolls. GCF was sampled with a filter paper strip (PerioPaper, Oraflow, Amityville, NY). Paper strips were carefully inserted into the crevice for 30 seconds. Strips contaminated with blood were discarded. The absorbed GCF volume of each strip was recorded by a pre-calibrated electronic device (Periotron 8000, Oraflow). The readings from the Periotron 8000 were converted to an actual volume (µl) by refence to standard curve. The collected paper strips were directly placed in microcentrifuge tubes and stored at -80°C until laboratory analysis.

*Collection and processing of subgingival plaque samples*

Approximately 15 minutes after GCF collection, subgingival plaque samples were collected from the same sites as GCF, using two standardized ♯30 sterile paper points as described earlier (Belibasakis et al. 2014). One point was inserted at a 45° angle and the other parallel to the long axis of the tooth and left in place for 10 sec. The paper points were then frozen at −80°C until further use.

*Subgingival plaque and saliva sample preparation for amplicon sequencing*

Saliva samples were thawed and centrifuged at 13000 rpm for 10 min, supernatants discarded and the pellet resuspended in 200 µl sterile Tris EDTA buffer. The resuspended saliva sample was added to a well, in a 96-deepwell-plate. For the subgingival plaque samples, the paperpoints were pooled per subject, and added to a well in a 96-deepwell plate. Each well contained 200 µl Tris-saturated phenol, 250 µl 0.1 mm zirconium beads and 100 µl lysis buffer. For dental plaque samples, 200 µl of Tris EDTA buffer was added. Samples were mechanically lysed by bead-beating at 1200 rpm for 2 min and DNA was isolated with the Mag MiniKit (LGC Genomics, Berlin, Germany, Mag mini kit). Bacterial DNA concentration was determined by quantitative polymerase chain reaction (qPCR) using a universal primer probe set targeting the 16S rRNA gene (Ciric et al. 2010).

*Control samples*

To control for potential bacterial DNA contaminants, both unused sterile paperpoints and blank controls with reagents alone were subjected to DNA isolation and included in all subsequent steps of sample sequencing. With each PCR batch at least one negative PCR blank control containing a PCR grade water instead of sample DNA was included. Additionally to the negative controls, a positive control – a commercially available Mock Community sample containing equimolar amount of DNA from 20 bacterial isolates (HM-782, BEI Resources, Manassas, VA, USA) was included.

*16S rDNA sequencing*

Next, 1 ng of DNA was used to amplify the V4 hypervariable region of the 16S rRNA gene, as described previously (Kozich et al. 2013), except that 30 amplification cycles were performed. The amplicons were pooled equimolarly and purified from agarose gel (Illustra™, GE Healthcare, Little Chalfont, United Kingdom). All controls added to the final amplicon pool. Paired-end reads of 251 bp were generated by paired-end sequencing of the amplicons using the Illumina MiSeq platform and Illumina MiSeq reagent kit V3 (Illumina, Inc., San Diego, CA) at the Tumor Genome Analysis Core (TCGA) of UAMC (Amsterdam, The Netherlands). The flowcell was loaded with 12 pmol DNA containing 25% Phix.

*Sequencing data processing*

The paired-end reads were merged, quality-filtered and clustered into operational taxonomic units (OTUs) at 97% similarity as described previously (Koopman et al. 2016). However, a maximum of 25 mismatches (10%, as the current reads were 2 × 251 nt long) was allowed during read merging, before quality filtering at 0.5 maximum expected error. The most abundant sequence of each OTU was classified using the RDP classifier (Wang et al. 2007) (min. confidence 0.8) and the Human Oral Microbiome Database version 14.51 (Chen et al. 2010).

*Assessment of salivary antimicrobial peptides*

Saliva samples were centrifuged for 30 min at 1000 rpm at 4°C (Eppendorf Thermomixer). ELISA Hu-HNP1-3 and ELISA Hu-LL-37 (Hycult Biotech) was performed according to the instructions of the manufacturer with the following modifications: incubation time was increased to 2 h and samples were diluted 1:200 and 1:10.

*Cytokine profiling by multiplex assay*

On the day of analysis, four GCF samples were pooled and eluted in 420 μl of phosphate-buffered saline (pH 7.2), by centrifugation at 5000×g for 10 min at 4°C. The cytokine 30-Plex panel (Novex®, ThermoFisher Scientific, USA) was used to quantify collected supernatants from GCF and saliva on the Luminex®200 platform C as described earlier (Afacan et al. 2018). The panel consisted of nineteen cytokines: G-CSF, GM-CSF, IFN-α, IFN-γ, IL-1β, IL-1RA, IL-2, IL-2R, IL-4, IL-5, IL-6, IL-7, IL-8, IL-10, IL-12 (p40/p70), IL-13, IL-15, IL-17, TNF-α; seven chemokines: Eotaxin, CXCL10 (IP 10), MCP-1, MIG, MIP-1α, MIP-1β, RANTES and four growth factors: EGF, FGF-basic, HGF, VEGF. The levels of the detected analytes were reported as pg/ml.

*Statistical analyses*

Differences in nominal variables were assessed by Pearson chi-square test. Normality of the single-variate scaled data was assessed using Kolmogorov-Smirnov normality test. Unrelated data between the groups (SCN patients vs controls) was compared using either Independent Samples T-test or Mann-Whitney test. Related data (SCN baseline vs 6-month follow-up) was compared using either paired samples T-test or Wilcoxon Signed Ranks test. Significance level was set at 0.05. The above analyses were performed using SPSS version 25.

For data ordination in principal coordinates (PCA), both microbial profile data and immunological data were normalized by log-2 transformation, while the differences in microbial or immunological profiles were assessed using permutational analysis of variance (PERMANOVA) with Bray-Curtis similarity, using PAST software (Hammer et al. 2001).

To identify discriminatory OTUs or genera, the linear discriminant analysis effect size (LEfSe) biomarker discovery tool (Segata et al. 2011) was used. Both for the OTU-level and genus-level analysis, only OTUs or genera that contributed to at least 0.01% of the total reads, were included.

To assess associations between the immunological parameters and microbiome, Spearman correlation was performed in R (version 3.6.0) on each immunological parameter and OTUs present in each group at a minimum abundance of 0.1%, significance was set at p<0.05, without correction for multiple comparisons. Only OTUs that had at least one significant correlation with at least one immunological parameter in at least one of the groups were further analyzed using TM4 (Saeed et al. 2006) of Multiple Array Viewer MEV version 4.9.0 and significant differences among groups assessed using multi-class Significance Analysis of Microarrays (SAM), set at 0% false discovery.

**References:**

Afacan B, Öztürk VÖ, Geçgelen Cesur M, Köse T, Bostanci N. 2018. Effect of orthodontic force magnitude on cytokine networks in gingival crevicular fluid: A longitudinal randomized split-mouth study. European Journal of Orthodontics. 41(2):214-222.

Belibasakis GN, Öztürk V-Ö, Emingil G, Bostanci N. 2014. Soluble triggering receptor expressed on myeloid cells 1 (strem-1) in gingival crevicular fluid: Association with clinical and microbiologic parameters. Journal of Periodontology. 85(1):204-210.

Bostanci N, Öztürk VÖ, Emingil G, Belibasakis GN. 2013. Elevated oral and systemic levels of soluble triggering receptor expressed on myeloid cells-1 (strem-1) in periodontitis. Journal of Dental Research. 92(2):161-165.

Chen T, Yu W-H, Izard J, Baranova OV, Lakshmanan A, Dewhirst FE. 2010. The human oral microbiome database: A web accessible resource for investigating oral microbe taxonomic and genomic information. Database. 2010.

Ciric L, Pratten J, Wilson M, Spratt D. 2010. Development of a novel multi-triplex qpcr method for the assessment of bacterial community structure in oral populations. Environ Microbiol Reports. 2(6):770-774.

Hammer O, Harper DAT, Ryan PD. 2001. Past: Paleontological statistics software package for education and data analysis Palaeontol Electr. 4:1-9.

Koopman JE, Buijs MJ, Brandt BW, Keijser BJF, Crielaard W, Zaura E. 2016. Nitrate and the origin of saliva influence composition and short chain fatty acid production of oral microcosms. Microbial Ecology. 72(2):479-492.

Kozich JJ, Westcott SL, Baxter NT, Highlander SK, Schloss PD. 2013. Development of a dual-index sequencing strategy and curation pipeline for analyzing amplicon sequence data on the miseq illumina sequencing platform. Applied and Environmental Microbiology. 79(17):5112-5120.

Saeed AI, Bhagabati NK, Braisted JC, Liang W, Sharov V, Howe EA, Li J, Thiagarajan M, White JA, Quackenbush J et al. 2006. Tm4 microarray software suite. Methods in enzymology. Academic Press. p. 134-193.

Segata N, Izard J, Waldron L, Gevers D, Miropolsky L, Garrett W, Huttenhower C. 2011. Metagenomic biomarker discovery and explanation. Genome Biology. 12(6):R60.

Wang Q, Garrity GM, Tiedje JM, Cole JR. 2007. Naïve bayesian classifier for rapid assignment of rrna sequences into the new bacterial taxonomy. Applied and Environmental Microbiology. 73(16):5261-5267.

Yılmaz Karapınar D, Patıroğlu T, Metin A, Çalışkan Ü, Celkan T, Yılmaz B, Karakaş Z, Karapınar TH, Akıncı B, Özkınay F et al. 2019. Homozygous c.130–131 ins a (pw44x) mutation in the hax1 gene as the most common cause of congenital neutropenia in turkey: Report from the turkish severe congenital neutropenia registry. Pediatric Blood & Cancer. 66(10):e27923.

**Supplementary results**

*Study Population characteristics*

In this study, 10 clinically severe congenital neutropenia (SCN) patients (9 females), aged 5-23 years (mean 11.1, SD 6.6) from different parts of Turkey were enrolled (Table 1) and compared with 12 healthy gender-matched controls (aged 5-22 years, mean 11.5, SD 4.8, 11 females). There was no significant difference in age between the groups (p>0.05). All SCN patients fulfilled the diagnostic criteria. The median duration of neutropenia was 52 months, ranging from 36 to 168 months. The median age at the onset of symptoms was 4 months (1–11 months). The median age at time of diagnosis was 19 months (2–41 months). The median diagnosis lag between the onset of symptoms and the medical visit was 12 months (1–36 months). A total 3 of the 10 patients were diagnosed before the age of 12 months. The median follow-up period for all patients was 68 months (range 29-138 months). None of them developed hematologic malignancy or sepsis requiring hospitalization during the G-CSF treatment period.

Family history indicated that 4 out of 9 families had first degree cousin marriage. Patients 109 and 110 were siblings. Four of the patients had homozygous HAX1 mutation, two patients had biallelic CSF3R mutation, one - ELANE mutation, while one patient with Glycogen Storage Disease type 1b was not evaluated for SCN gene mutations (Table 1).

All patients had a history of recurrent infections, the frequency of infections varied among patients with a median of 6 times a year (range 4–8 times a year) before the G-CSF therapy and twice a year (0–4) after the G-CSF treatment was started. Most patients suffered from respiratory infections, with frequent upper airway infection in almost all cases. Among the remaining infections, otitis media was the most common (4/10 cases), followed by purulent skin infections (2 cases), enteritis (1 case). Hepatosplenomegaly was observed in the patient with GSD type1b.

- At the diagnosis, before the start of the G-CSF therapy, median absolute neutrophil counts (ANC) were 320 (112-440) x10^6^/l, median eosinophil counts - 780 (420-1100) x10^6^/l and median monocyte counts - 1330 (800-2200) x10^6^/l, and median white blood cell counts - 5600 (3500-11200) x10^6^/l (Supplementary Table 1). ANC was followed to raise neutrophils to approximately 1000x10^6^/l, resulting in adjustment of G-CSF.

Giemsa staining of bone marrow aspiration and biopsy samples of the cases showed decreased myeloid cell counts with a maturation arrest of neutrophil precursors at the promyelocytic stage of differentiation, with an increment of monocyte and eosinophil counts in 5 patients. In three patients, maturation arrest was seen in later stages of myeloid lineages. There was no maturation arrest in two patients (101 and 103). Erythroid cells, lymphocytes and megakaryocytes were normal in number and morphology in samples from all patients.

*Salivary antimicrobial peptides and cytokine profiles*

Among the immunological parameters measured in saliva (Supplementary Table 3), granulocyte-macrophage colony stimulating factor (GM-CSF) was significantly higher in the test group at the baseline (p=0.007) and at the 6-month recall (p=0.028) than in the saliva of the control group. The same was observed for IL-1RA (p=0.005 and p=0.019). IL-4 was significantly higher at the baseline in the test group (p=0.028) than in the control group. The same was observed for IL-7 (p=0.011).

Monokine induced by IFN-γ (MIG) (p=0.038), IL-5 (p=0.05), interferon gamma-induced protein 10 (IP-10) (p=0.008) and monocyte chemoattractant protein-1 (MCP-1) (p=0.021) and all decreased significantly between the baseline and the 6-month recall visit in the test group.

*GCF volume and cytokine profiles*

GCF collected in SCN-patients at the baseline had a significantly higher concentration of G-CSF compared to the control (p=0.021) (Supplementary Table 4). The same was observed for GM-CSF (p=0.002), IFN-α (p=0.009), IFN-γ (p=0.025), IL-1RA (p=0.009), IL-6 (p=0.025), IL-8 (p=0.036), IL-10 (IL-10) (p=0.021), IL-13 (p=0.003), IL-15 (p=0.030), IL-17 (p=0.021), TNF-α (p=0.005), Eotaxin (p=0.05), IP-10 (p=0.006), MCP-1 (p=0.036), MIG (p=0.03), MIP-1α (p=0.014), MIP-1β (p=0.017), RANTES (p=0.014), FGF-basic (p=0.021) and VEGF (p=0.006).

IL-1β was significantly higher in the baseline samples of the test group compared to the control (p=0.003) and it decreased significantly between the baseline and the 6-month follow-up (p=0.008). The same was observed for IL-2 (p=0.026 and p=0.044), EGF (p=0.017 and p=0.015) and HGF (p=0.038 and p=0.009). IL-4 decreased significantly between the baseline and the 6-month recall (p=0.005).

*Samples for microbiome composition*

Subgingival plaque samples from 12 controls, 10 SCN baseline and 9 - 6-month recall samples (all but 107), and saliva samples from 11 controls (all but 209), 8 SCN baseline (all but 103, 107) and 9 –6-month recall samples (all but 107) were available for sequencing. After sequencing, one saliva sample (control, 208) did not yield sufficient reads and was excluded from the analyses.

**Supplementary figures**


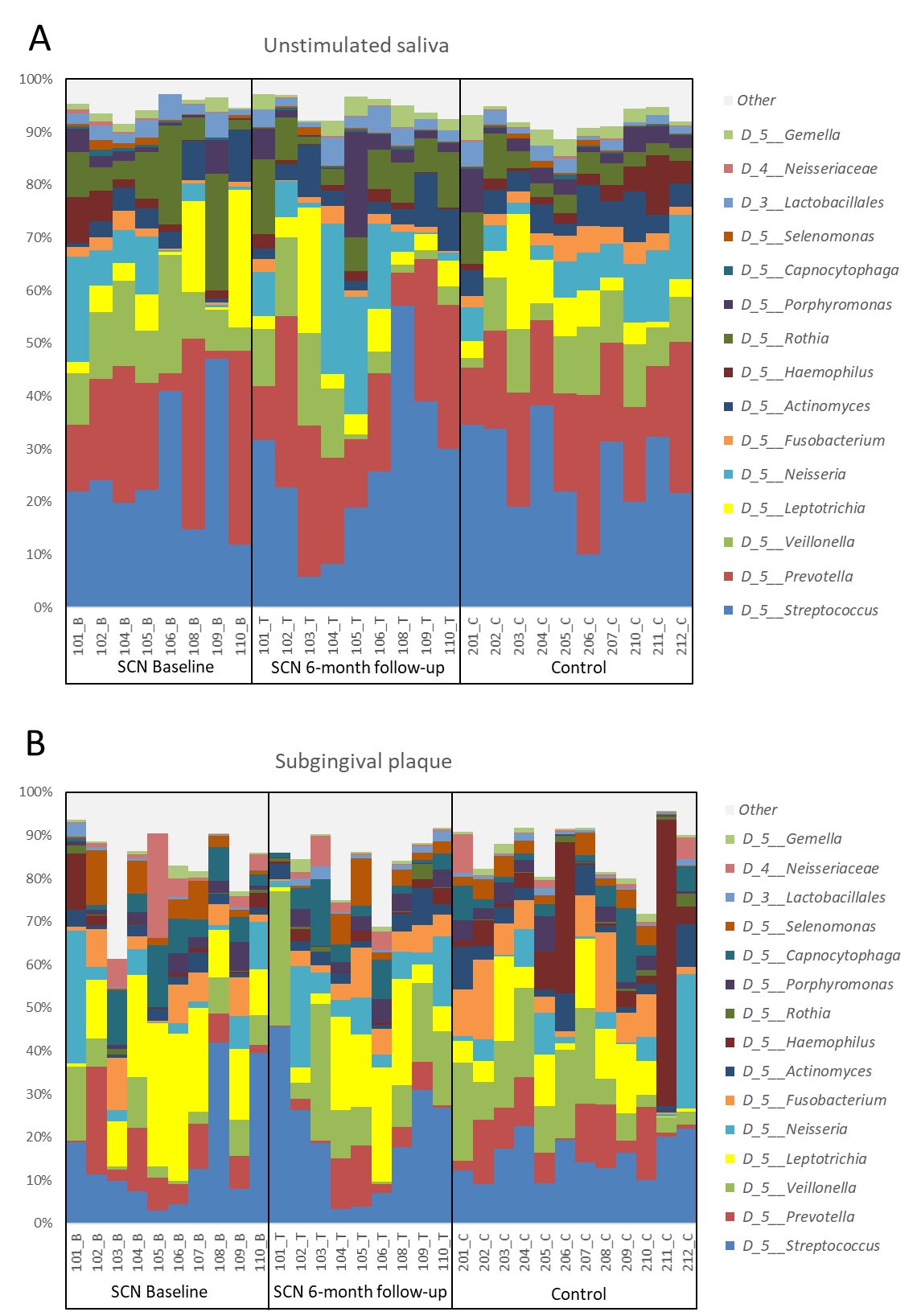


**Supplementary Figure 1.** Relative abundance of top 15 genera or higher taxa in saliva (A) and subgingival plaque (B) of individual subjects. The remaining taxa are summed up as Other.


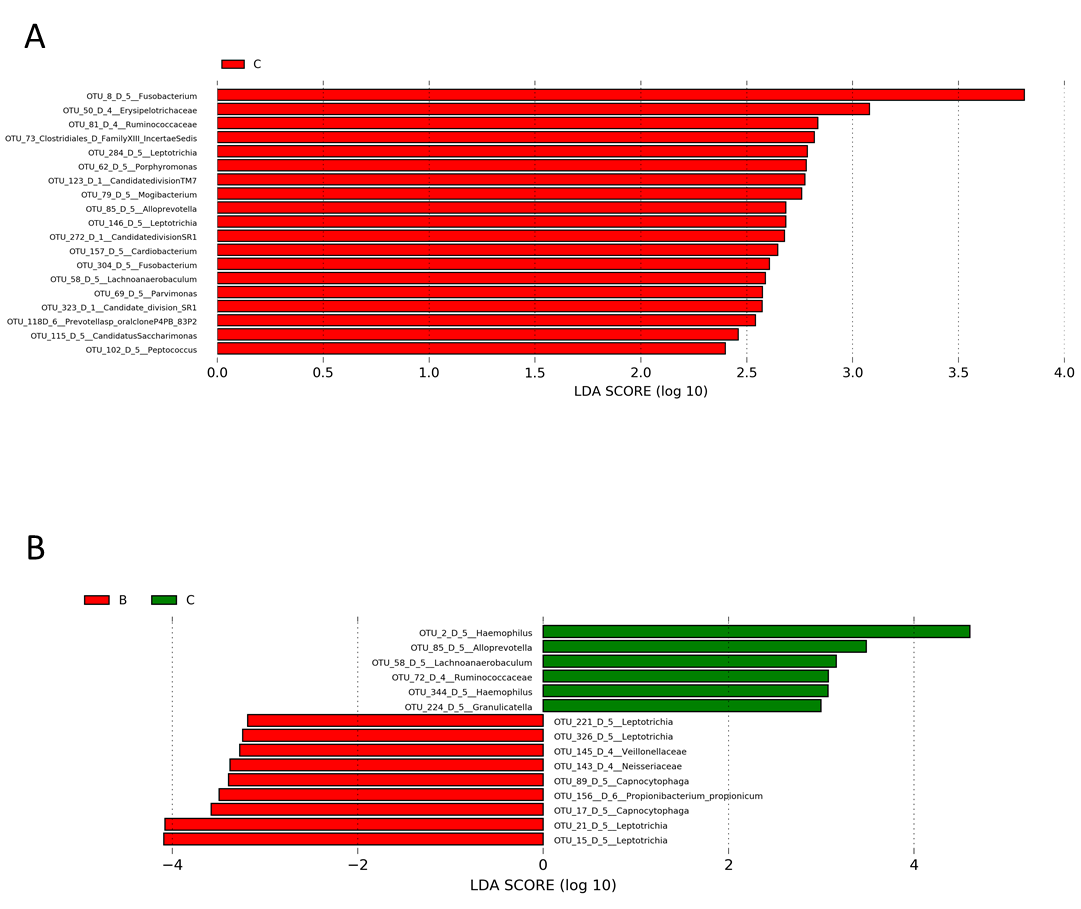


**Supplementary Figure 2.**  Significantly discriminatory OTUs in A) saliva and B) subgingival plaque between the SCN baseline samples and the controls as identified by LEfSe. SCN baseline – B, Control group – C.

**Supplementary tables**

**Supplementary Table 1.** Blood counts and immunoglobulin values in SCN patients at the diagnosis, before the start of G-SCF therapy.

| **SCN patient** | **WBC**  **(cells/mm^3^)** | **ANC**  **(cells/mm^3^)** | **AMoC (cells/ mm^3^)** | **AEoC**  **(cells/**  **mm^3^)** | **Hb**  **(g/dl)** | **Htc**  **(%)** | **MCV**  **(fL)** | **PLT**  **(cells/ mm^3^)** | **IgG**  **(mg/dl)** | **IgM**  **(mg/dl)** | **IgA**  **(mg/dl)** | **IgE**  **(mg/dl)** |
| --- | --- | --- | --- | --- | --- | --- | --- | --- | --- | --- | --- | --- |
| 101 | 4640 | 350 | 800 | 870 | 12.6 | 38 | 82.4 | 232000 | 1210 | 78 | 124 | 16 |
| 102 | 4170 | 373 | 1170 | 420 | 11.7 | 39 | 81 | 283000 | 804 | 112 | 107 | 69 |
| 103 | 9900 | 413 | 1360 | 960 | 12 | 38 | 79 | 534000 | 1100 | 121 | 118 | 28 |
| 104 | 3500 | 290 | 920 | 790 | 15.4 | 44.8 | 82 | 240000 | 1250 | 88 | 191 | 369 |
| 105 | 5600 | 112 | 1440 | 690 | 11.8 | 35 | 89 | 402000 | 2250 | 93.7 | 133 | 110 |
| 106 | 5910 | 167 | 1580 | 770 | 11.5 | 35 | 80 | 433000 | 1280 | 130 | 103 | 11 |
| 107 | 5050 | 440 | 880 | 640 | 9.4 | 31 | 74 | 423000 | 1100 | 115 | 96 | 45 |
| 108 | 5600 | 350 | 1300 | 510 | 11.6 | 35 | 78 | 412000 | 1200 | 98 | 65 | 44 |
| 109 | 11200 | 140 | 2200 | 1100 | 10.9 | 31 | 75 | 245000 | 1700 | 177 | 219 | 42 |
| 110 | 7400 | 190 | 2000 | 1010 | 11.5 | 34 | 81 | 330000 | 1100 | 89 | 68 | 61 |

WBC: White Blood Cell, ANC: Absolute neutrophil count, AMoC: Absolute monocyte count, AEoC: Absolute Eosinophil count, Hb: Hemoglobin, Htc: Hematocrit, MCV: Mean Corpuscular volume, PLT: Platelet count

**Supplementary Table 2.** Clinical oral parameters of severe congenital neutropenia (SCN) and control groups.

|  | **SCN (baseline)** | | **SCN (6 months)** | | **Control** | |
| --- | --- | --- | --- | --- | --- | --- |
|  | **Mean± SD** | **Median (min-max)** | **Mean± SD** | **Median (min-max)** | **Mean± SD** | **Median (min-max)** |
| Age (years) | 11.1±6.6 | 7.5 (5-23) |  |  | 11.5±4.8 | 11 (5-22) |
| dmft | 4.1±2.7 | 4 (0-9) | NA | NA | 3.4±2.0 | 3 (1-8) |
| DMFT | 3.4±3.6 | 1.5 (0-8) | NA | NA | 1.9±1.5 | 2.0 (0-5) |
| PPD (mm) | 1.9±0.3 | 1.9 (1.4-2.6) | 2.0±0.4 | 1.9 (1.5-2.8) | 1.9±0.5 | 1.8 (1.4-2.9) |
| CAL (mm) | 1.9±0.3 | 1.9 (1.4-2.6) | 2.0±0.4 | 1.9 (1.5-2.8) | 1.9±0.5 | 1.8 (1.4-2.9) |
| BOP (%) | 31±15 | 30 (15-55) | 17±6 | 15 (8-28)*^$^ | 24±9 | 23 (15-42) |
| PI (%) | 65±16 | 66 (38-88) | 25±6.7 | 24 (16-38)*^$^ | 68±13 | 72 (45-85) |

* - Significantly different from the baseline (p<0.05, Wilcoxon Signed Ranks test)

^$^ - significantly different from the control group (p<0.05, Mann-Whitney test)

**Supplementary Table 3.** Antimicrobial peptides and immunological parameters in saliva.

|  | **SCN baseline**  mean±SD (median, range) | **SCN 6 months**  mean±SD (median, range) | **Control**  mean±SD (median, range) |
| --- | --- | --- | --- |
| **Antimicrobial peptides:** | | | |
| Hu-HPN1-3 (ng/ml) | 688±502 (599, 184-1822) | 775±476 (661, 305-1505) | 775±592 (846, 21-1910) |
| LL-37 (ng/ml) | 16.5±21 (6.5, 1-66) | 16.6±20 (9.5, 1.8-67) | 12.89 (12, 2-29) |
| **Cytokines:** | | | |
| G-CSF (pg/ml) | 175±60 (155, 122-309) | 235±131 (192, 114-538) | 150±43 (152, 64-226) |
| GM-CSF (pg/ml) | 4.2±4.9 (2.7, 1.3-17) ^$^ | 2.6±1.2 (2.4, 0.9-4) ^$^ | 1.4±0.6 (1.2, 0.8-2.8) |
| IFN-α (pg/ml) | 60±6 (61, 50-66) | 59±6 (61, 46-64) | 52±13 (59, 21-62) |
| IFN-γ (pg/ml) | 30±12 (30, 16-46) | 28±11 (33, 11-38) | 28±17 (27, 10-71) |
| IL-1β (pg/ml) | 138±177 (81, 48-606) | 129±167 (43-567) | 68±23 (71, 23-97) |
| IL-1RA (ng/ml) | 304±101 (306, 193-517)^ | 297±112 (301, 131-477)^ | 180±84 (175, 27-289) |
| IL-2 (pg/ml) | 10±0.9 (9.7, 8.5-11) | 8.9±1.5 (9, 5.7-10.5) | 8.6±2.5 (8.4, 4.4-12) |
| IL-2R (pg/ml) | 107±28 (114, 69-139) | 95±27 (105, 44-122) | 102±41 (103, 41-194) |
| IL-4 (pg/ml) | 27±3.7 (28, 21-31)^ | 25±3.7 (24, 19-31) | 22±5.6 (21, 13-29) |
| IL-5 (pg/ml) | 2.3±0.6 (2.5, 1.4-2.9) ^$^ | 1.8±0.5 (1.9, 0.7-2.2) | 2.2±1 (2.3, 0.3-3.4) |
| IL-6 (pg/ml) | 100±71 (65, 37-245) | 81±32 (67, 42-130) | 75±55 (47, 17-179) |
| IL-7 (pg/ml) | 45±22 (39, 6.5-79)^$^ | 33±22 (43, 0.8-58) | 22.7±15 (20, 2.9-51) |
| IL-8 (ng/ml) | 4±4 (2, 0.8-14) | 2.9±2.3 (2.3, 0.5-6.6) | 3±3.3 (1.8, 0.05-10) |
| IL-10 (pg/ml) | 2.2±0.9 (2.1, 1-3) | 2±0.7 (1.8, 1-3.3) | 2±0.93 (1.8, 0.7-3.8) |
| IL-12 (pg/ml) | 66±9 (70, 54-78) | 66±7 (65, 56-79) | 67±22 (65, 23-99) |
| IL-13 (pg/ml) | 30±6 (29, 22-38) | 27±4.6 (27, 21-34) | 27±7 (28, 12-36) |
| IL-15 (pg/ml) | 73±15 (72, 50-98) | 68±14 (72, 36-80) | 63±20 (62, 21-95) |
| IL-17 (pg/ml) | 3.4±1.5 (3.2, 0.2-5.2) | 2.8±1.7 (3.2, 0.2-5.2) | 2.7±1.5 (2.7, 0.8-5.2) |
| TNF-α (pg/ml) | 5.6±1.3 (5.4, 3.4-7.5) | 6±1.9 (6.3, 2.5-9.3) | 4.7±1.2 (5.2, 2.7-6.2) |
| **Chemokines:** | | | |
| Eotaxin (pg/ml) | 1.2±1.5 (0.2, 0.2-4.2) | 1.2±1.2 (0.7, 0.2-3.3) | 1.1±2.2 (0.2, 0.2-7) |
| CXCL10 (IP-10) (pg/ml) | 56±144 (6, 4.5-440)* | 6.9±7 (4.6, 4-25) | 28±28 (28, 2.7-82) |
| MCP-1 (pg/ml) | 428±139 (400, 209-677)* | 346±67 (355, 205-439) | 572±472 (371, 62-1446) |
| MIG (pg/ml) | 690±1107 (111, 48-3313)* | 118±96 (85, 43-338) | 777±1059 (313, 2-3116) |
| MIP-1α (pg/ml) | 43±25 (35, 26-106) | 44±28 (34, 30-117) | 37±15 (35, 21-75) |
| MIP-1β (pg/ml) | 46±12 (40, 33-70) | 44±13 (41, 26-74) | 42±15 (39, 12-67) |
| RANTES (pg/ml) | 2.9±5.4 (0.6, 0.6-17) | <0.6^#^ | 3±7.7 (0.6, 0.6-25) |
| **Growth factors:** | | | |
| EGF (pg/ml) | 388±172 (319, 208-756) | 372±170 (305, 174-763) | 389±329 (309, 23-1119) |
| FGF-basic (pg/ml) | 21±2.4 (20, 18-25) | 20±2.6 (20, 16-24) | 20±5.8 (20, 11-32) |
| HGF (pg/ml) | 116±16 (122, 92-145) | 108±24 (114, 59-142) | 101±32 (94, 40-142) |
| VEGF (pg/ml) | 180±31 (166, 131-216) ^$^ | 173±26 (166, 130-215) | 172±48 (184, 52-222) |

^#^ - below detection limit, 0.59 pg/ml

* - Significantly different from the 6-month recall (p<0.05, Wilcoxon Signed Ranks test)

^$^ - significantly different from the control group (p≤0.05, Mann-Whitney test)

^^^ - significantly different from the control group (p<0.05, Independent Samples T-test)

**Supplementary Table 4.** Immunological parameters in gingival crevicular fluid (GCF).

|  | **SCN baseline**  mean±SD (median, range) | **SCN 6 months**  mean±SD (median, range) | **Control**  mean±SD (median, range) |
| --- | --- | --- | --- |
| **Cytokines:** | | | |
| G-CSF (pg/ml) | 49±10 (50, 31-62) ^$^ | 43±17 (48, 14-60) | 39±10 (41, 15-51) |
| GM-CSF (pg/ml) | 0.8±1.7 (0.2, 0.04-5.4) ^$^ | 0.2±0.12 (0.14, 0.04-0.4) | 0.13±0.1 (0.14, 0.03-0.23) |
| IFN-α (pg/ml) | 16±3 (17, 9-20) ^$^ | 15±5 (15.6, 4-20) | 13±2.7 (12, 7.5-17) |
| IFN-γ (pg/ml) | 17±3.2 (18, 9-21) ^$^ | 14±7.3 (15, 0.3-21) | 14±4 (14, 7.5-20) |
| IL-1β (pg/ml) | 24±10 (23, 9-42) ^$^* | 16±7.5 (15, 4-25) | 14±3.3 (13, 8-19) |
| IL-1RA (ng/ml) | 34±18 (30, 8-64)^ | 29±24 (19, 2-71) | 18±9 (16, 6-37) |
| IL-2 (pg/ml) | 5±2 (5.4, 0.7-7)^^#^ | 4.2±2.5 (4.3, 0.3-7) | 3.4±1.4 (3.2, 1-5) |
| IL-2R (pg/ml) | 33±9.7 (35, 16-44) | 25±14 (21, 1-44) | 27±11 (28, 4.7-42) |
| IL-4 (pg/ml) | 25±5 (26, 18-31)^#^ | 22±7 (24, 8-30) | 21±5.8 (21, 12-29) |
| IL-5 (pg/ml) | 0.8±0.6 (0.5, 0.4-2) | 1±0.8 (0.4, 0.4-2.6) | 0.6±0.4 (0.5, 0.4-1.7) |
| IL-6 (pg/ml) | 7.2±3.4 (6.4, 2.6-13.8) ^$^ | 4.7±2.3 (5, 0.8-7.8) | 4.7±1.5 (5, 2-6.5) |
| IL-7 (pg/ml) | 9.8±2.4 (9.6, 5.6-13.4) | 9.5±5.2 (8.4, 4-17) | 8.9±2.6 (9.3, 2.9-11.8) |
| IL-8 (pg/ml) | 939±699 (893, 86-2396) ^$^ | 560±457 (480, 26-1350) | 484±310 (486, 35-1153) |
| IL-10 (pg/ml) | 0.32±0.1 (0.35, 0.18-0.49) ^$^ | 0.21±0.04 (0.2, 0.14-0.28) | 0.24±0.1 (0.2, 0.14-0.42) |
| IL-12 (pg/ml) | 19±4.3 (19, 13-26) | 19±5 (20, 11-25) | 16±3.4 (17, 9-21) |
| IL-13 (pg/ml) | 12±2 (12, 8-14) ^$^ | 9±5.3 (11, 0.6-14) | 8.6±3.1 (8.9, 0.6-12) |
| IL-15 (pg/ml) | 17±5 (18, 9-22) ^$^ | 13±8 (16, 2-23) | 12±5 (11, 2.6-18) |
| IL-17 (pg/ml) | 1.8±0.7 (1.7, 1-2.7) ^$^ | 1.4±0.7 (1, 1-2.8) | 1.2±0.4 (1, 1-2.1) |
| TNF-α (pg/ml) | 3.6±1 (3.6, 2-5)^ | 2.9±1.6 (2.8, 0.9-5) | 2.5±0.9 (2.4, 1.4-4) |
| **Chemokines:** | | | |
| Eotaxin (pg/ml) | 1.3±0.7 (1.3, 0.2-2.4) ^$^ | 0.9±0.6 (1.1, 0.2-1.6) | 0.7±0.6 (0.6, 0.2-1.9) |
| IP-10 (pg/ml) | 3.4±1.3 (3, 1.5-6) ^$^ | 2.5±1.2 (2.6, 0.4-4) | 2.4±0.7 (2.6, 0.9-3) |
| MCP-1 (pg/ml) | 178±54 (183, 87-263) ^$^ | 131±55 (143, 38-195) | 134±38 (148, 55-185) |
| MIG (pg/ml) | 40±16 (38, 21-72) ^$^ | 36±11 (38, 25-52) | 29±6.8 (29, 21-41) |
| MIP-1α (pg/ml) | 20±15 (15, 8-58) ^$^ | 14±9 (16, 0.9-27) | 11±3 (12, 3-16) |
| MIP-1β (pg/ml) | 36±34 (23, 6-119) ^$^ | 23±15 (23, 3.8-49) | 16±5 (18, 5-22) |
| RANTES (pg/ml) | 5.2±2.3 (6.3, 1-8) | 3.5±1.7 (4.2, 1-5.5) | 3.2±1.1 (3.1, 2-5) |
| **Growth factors:** | | | |
| EGF (pg/ml) | 14±5 (14, 7-23) ^$^* | 9.8±5 (11, 0.3-14) | 11±1.4 (11, 8-13) |
| FGF-basic (pg/ml) | 14±3.3 (14, 7-18) ^$^ | 12.5±5 (15, 4-17) | 12±2.2 (12, 8-16) |
| HGF (pg/ml) | 82±22 (90, 37-105) ^$^* | 66±28 (67, 22-99) | 60±17 (62, 22-80) |
| VEGF (pg/ml) | 38±8 (39, 21-47) | 32±15 (31, 6-47) | 31±5.7 (32, 17-38) |

* - significantly different from the 6-month recall (p<0.05, Wilcoxon Signed Ranks test)

^$^ - significantly different from the control group (p≤0.05, Mann-Whitney test)

^#^ - significantly different from the 6-month recall (p<0.05, Paired Samples T-test test)

^^^ - significantly different from the control group (p<0.05, Independent Samples T-test)
